# Supplementary material for: Collective buoyancy-driven dynamics in swarming enzymatic nanomotors
Source: Nat Commun. 2024 Oct 29;15:9315. doi: 10.1038/s41467-024-53664-w (PMC11522643; doi:10.1038/s41467-024-53664-w)
Supplement: Supplementary file 1 — Supplementary Information [file 41467_2024_53664_MOESM1_ESM.pdf]

Supplementary Information

Collective Buoyancy-driven Dynamics in Swarming Enzymatic Nanomotors

Shuqin Chen[1,2], Xander Peetroons[1], Anna C. Bakenecker[1], Florencia Lezcano[1], Igor S Aranson[3,4,5*], Samuel Sánchez[1,6*]

[1]Institute for Bioengineering of Catalonia (IBEC), The Barcelona Institute for Science and Technology (BIST), Baldiri i Reixac 10-12, Barcelona 08028, Spain

[2]Faculty of Physics, University of Barcelona, Martí i Franquès 1, Barcelona 08028, Spain

[3]Departments of Biomedical Engineering, The Pennsylvania State University, University Park, Pennsylvania 16802, United States

[4]Departments of Chemistry, The Pennsylvania State University, University Park, Pennsylvania 16802, United States

[5]Departments of Mathematics, The Pennsylvania State University, University Park, Pennsylvania 16802, United States

[6]Catalan Institute for Research and Advanced Studies (ICREA), Passeig Lluís Companys 23, Barcelona 08010, Spain

Corresponding authors:

*Igor S Aranson: isa12@psu.edu

*Samuel Sánchez: ssanchez@ibecbarcelona.eu

Supplementary Note 1. Solutal convection equations

We consider the following model: colloidal particles coated by the enzyme (urease) react with the fuel (urea). As a result of the enzymatic reaction, ammonia gas is released. The gas dissolves in the solvent (water), making it effectively lighter. Lighter volumes of solvent raise upward, creating convective plumes. The solvent flow entertains the colloidal particles (particulate) and brings them up. Once fuel is consumed, the reaction slows down, and the particulate sediments to the bottom. We neglect heat produced in the course of the reaction.

Our starting point is two-fluid hydrodynamics. One fluid will be a solvent with the dynamic viscosity η, velocity $\mathbf{v}$, and pressure p, and the solvent density ρ_0. Other fluid is the particulate with the volume density ρ, coarse-grained particulate velocity $\mathbf{u}$, pressure P. The particulate density ρ is $\rho = \rho_p \Phi$, where ρ_p is the density of a particulate particle, and Φ is the particulate particle filling fraction. The volume filling fraction Φ is related to the particle concentration n as $\Phi = V_0 n, V_0 = 4/3\pi a^3$ is the volume of a spherical particle of the radius a.

We also consider coupling to the chemical fluid field c that diffuses with the diffusion coefficient D_c and is consumed with the rate $\gamma\rho c$ due to the reaction between particulate and the fuel. The solvent is assumed to be incompressible, i.e., $\nabla\mathbf{v} = 0$. The chemical field which is of the form

$$\partial_t c + \nabla \cdot (\boldsymbol{v}c) = D_c \nabla^2 c - \gamma\rho c \tag{1}$$

This reaction-diffusion equation describes the advection of the fuel by the solvent flow $\mathbf{v}$, diffusion, and consumption due to chemical reaction with the particulate. The two-fluid hydrodynamics equations assuming that the particulate is dilute can be written in the following general form for the incompressible solvent:

$$\rho_0(\partial_t \mathbf{v} + \mathbf{v}\nabla\mathbf{v}) = \eta\nabla^2\mathbf{v} - \nabla p + \mathbf{z}_0\epsilon\rho c + \kappa n(\mathbf{u} - \mathbf{v}) \tag{2}$$

$$\nabla \cdot \mathbf{v} = 0 \tag{3}$$

Here η is the solvent dynamic viscosity, $\mathbf{z}_0\epsilon\rho c$ is the volume buoyancy force due to chemical reaction between particulate, $\mathbf{z}_0$ is the unit vector in the z-direction (z-axis is upward). The last term $\kappa n(\mathbf{u} - \mathbf{v})$ is due to the friction between particulate and the solvent (or drag force), κ is the friction coefficient. For spherical particles of the radius a, $\kappa = 6\pi\eta a$. Similar term will appear in the particulate equation:

$$\rho(\partial_t \mathbf{u} + \mathbf{u}\nabla\mathbf{u}) = \eta_\rho\nabla^2\mathbf{u} - \nabla P - \mathbf{z}_0\alpha g\rho + \kappa n(\mathbf{v} - \mathbf{u}) \tag{4}$$

$$\partial_t \rho + \nabla \cdot (\rho\mathbf{u}) = 0 \tag{5}$$

Since the particulate is assumed to be compressible, the pressure is to be determined from the equation of state $P = f(\rho)$. In the dilute limit, $P = q\rho, q$ is some coefficient that depends on temperature, etc. The term $\mathbf{z}_0 g\rho$ describes the sedimentation of the particulate,

g is the acceleration of gravity. Coefficient $\alpha = (\rho_p - \rho_0)/\rho_p$ includes the effect of the Archimedes force. While Eqs. (1)-(5) describe the system, they are cumbersome and hard to solve computationally. Therefore, we consider a simplification. We assume that the motion of particulate is over-damped and neglect the particulate acceleration $\rho(\partial_t \mathbf{u} + \mathbf{u}\nabla\mathbf{u})$ and viscous stress $\eta_p \nabla^2 \mathbf{u}$. The resulting equation is:

$$-\nabla P - \mathbf{z}_0 g\alpha\rho + \kappa n(\mathbf{v} - \mathbf{u}) = 0 \tag{6}$$

Substituting $\rho(\mathbf{v} - \mathbf{u})$ from Eq. (6) into the Navier-Stokes equation for the solvent, Eq. (2), we readily obtain

$$\rho_0(\partial_t \mathbf{v} + \mathbf{v}\nabla\mathbf{v}) = \eta\nabla^2\mathbf{v} - \nabla\tilde{p} - \mathbf{z}_0\rho(g\alpha - \epsilon c) \tag{7}$$

where $\tilde{p} = p + P$ is the re-normalized pressure. In the following, we drop tildes. Eq. (7) is identical to the Boussinesq model for the buoyancy-driven thermal convection. To exclude $\mathbf{u}$, it is useful to rewrite Eq. (6) in terms of the particulate density ρ rather than concentration n, $\rho = \rho_p V_0 n$:

$$-\nabla P - \mathbf{z}_0 g\alpha\rho + \kappa_1\rho(\mathbf{v} - \mathbf{u}) = 0 \tag{8}$$

where $\kappa_1 = \kappa/(\rho_p V_0) = 9\eta/(2\rho_p a^2)$. If we express the particulate velocity $\mathbf{u}$:

$$\mathbf{u} = \mathbf{v} - \frac{\dfrac{\nabla P}{\rho} + \mathbf{z}_0\alpha g}{\kappa_1} \tag{9}$$

and substitute into Eq. (5), we obtain:

$$\partial_t\rho + \nabla\cdot(\rho\mathbf{v}) = \frac{1}{\kappa_1}(\nabla^2 P + \alpha g\,\partial_z\rho) \tag{10}$$

Since in the dilute limit the pressure is $P = q\rho$, we obtain a reaction-diffusion equation:

$$\partial_t\rho + \nabla\cdot(\rho\mathbf{v}) = \frac{1}{\kappa_1}(q\nabla^2\rho + \alpha g\,\partial_z\rho) \tag{11}$$

To summarize the equations:

$$\rho_0(\partial_t \mathbf{v} + \mathbf{v}\nabla\mathbf{v}) = \eta\nabla^2\mathbf{v} - \nabla p - \mathbf{z}_0\rho(g\alpha - \epsilon c)$$

$$\partial_t c + \nabla\cdot(\mathbf{v}c) = D_c\nabla^2 c - \gamma\rho c$$

$$\partial_t\rho + \nabla\cdot(\rho\mathbf{v}) = \frac{1}{\kappa_1}(q\nabla^2\rho + \alpha g\,\partial_z\rho)$$

Supplementary Note 2. Depth-averaged description

To obtain the depth-averaged description, we will use the theoretical approach developed in Ref. [1] to describe experiments in Ref. [2]. We consider a cell where the height h is much

smaller than the lateral dimensions L. We integrate equation for vertical velocity v_z across the depth. It gives for the depth-averaged velocity $V = \int_0^h v_z \, dz/h$ the following equation:

$$\partial_t V = \nu \nabla_\perp^2 V - \eta V + \beta \langle \rho \tilde{c} \rangle - p(h)$$

Here $\langle \rho c \rangle \sim \tilde{\rho}, \tilde{c}$ are the depth averaged density and concentration, $\eta = 12\nu/h^2$. In the following, we drop tildes for briefty, $\nabla_\perp^2 = \partial_x^2 + \partial_y^2$. Here we make the following approximation: the vertical velocity, near the threshold is a symmetric function of z (flow between two parallel plates)

$$v_z(z) = \frac{3V}{2h^3}(h^2 - 4(z - h/2)^2)$$

Then, the horizontal velocity can be found from the incompressibility condition $\nabla \cdot \mathbf{v} = 0$. We assume that the particulate is always at the bottom part of the cells, between $0 < z < h/2$. Then, we are interested in only the horizontal velocity in the lower part of the cell, V_x, V_y. We assume that the horizontal velocity can be found from a potential $\mathbf{V}_\perp = \nabla_\perp \phi$. It gives the equation for the potential ϕ:

$$\nabla_\perp^2 \phi = -\int_0^{\frac{h}{2}} \partial_z \, v(z) dz = -\frac{3V}{2h} \qquad (12)$$

Note that the depth-averaged $\int_0^h \mathbf{v} \, \nabla \mathbf{v} dz = 0$ due to the symmetry. However, since we assume that the profiles of c and ρ are not symmetric, then $\int_0^{h/2} \mathbf{v} \, \nabla \rho dz \sim \mathbf{V}_\perp \nabla_\perp \rho \neq 0$, as well as the same equation for c. Finally, to implement a short-wave instability in the equation, we assume that pressure is given by the condition:

$$p = \int K(\mathbf{r} - \mathbf{r}') \tilde{\alpha} \rho c d\mathbf{r}'$$

where K is a kernel that should satisfy certain normalization conditions. It can be written in the Fourier space:

$$\widehat{K}(k) = \int \exp(i\mathbf{k}\mathbf{r}) K(\mathbf{r}) d\mathbf{r}$$

We assume that $\widehat{K}(k) = \exp(-\epsilon k^2)$ where parameter $\epsilon \sim h^2$ depended on the chamber height h. Combining this together, we obtain for the vertical velocity:

$$\partial_t V = \nu \nabla_\perp^2 V - \eta V + \beta \int K_1(\mathbf{r} - \mathbf{r}') \rho c d\mathbf{r}' - \mu V^3 \qquad (13)$$

where $\widehat{K}_1(k) = 1 - \exp(-\epsilon k^2)$. Note that $\widehat{K}_1(k = 0) = 0$: uniform distribution of particles does not produce any vertical flow since it is compensated by the vertical pressure gradient. The term $-\mu V^3$ is introduced to saturate the instability and prevent the blow-up of the solution. Finally, equations for ρ, c take form:

$$\partial_t c + \nabla_\perp(\mathbf{V}_\perp c) = D_c \nabla_\perp^2 c - \gamma \rho c \qquad (14)$$

$$\partial_t \rho + \nabla_\perp(\mathbf{V}_\perp \rho) = D_\rho \nabla_\perp^2 \rho \qquad (15)$$

Solving equations (13), (14), (15) and Eq. (12) generates the dynamics resembling the experiment.

Supplementary Figures

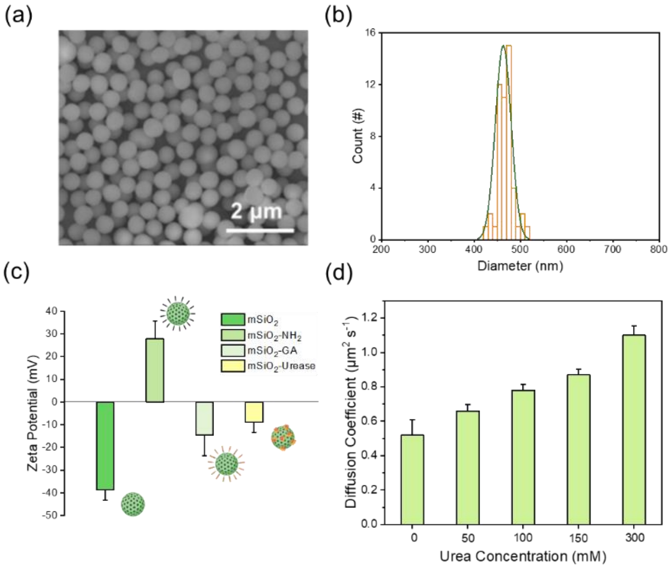

Supplementary Fig. 1 Synthesis and characterization of UrNMs. (a) SEM image of MSNPs. (b) Statistic particle size distribution of MSNPs. N=50. (c) Zeta potential changes during the process of MSNP surface modification. The data are shown as mean ± standard deviation (SD) of three independent experiments (N=3). The schematics were created in BioRender. Sánchez, S. (2023) BioRender.com/b39h124. (d) Diffusion coefficient measurement of UrNMs in elevated concentration of urea solutions by DLS. N=5.

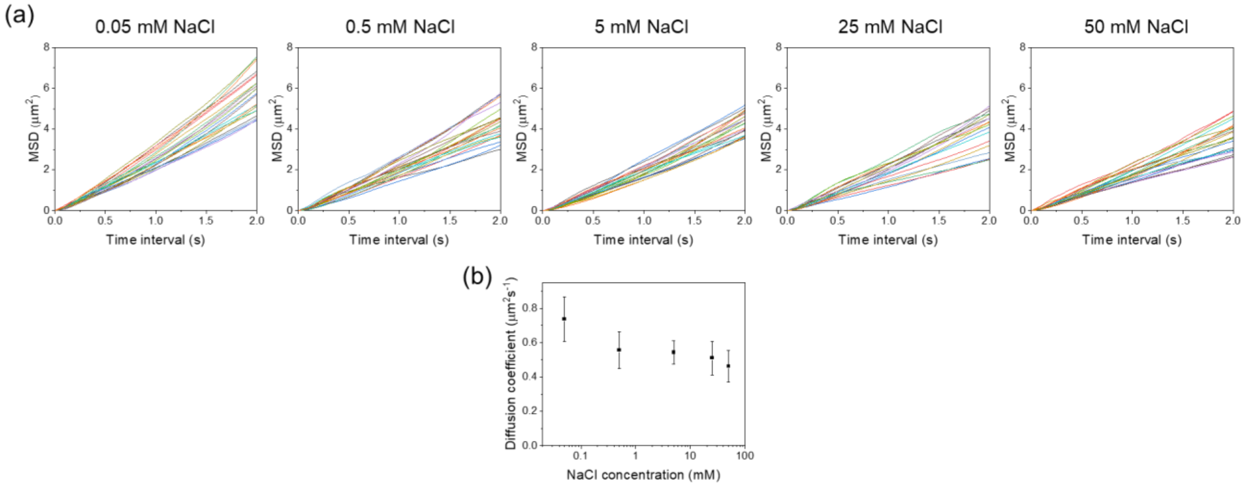

Supplementary Fig. 2 Motion analysis of UrNMs in urea with varying NaCl concentrations. (a) Mean square displacement (MSD) of UrNMs in 50 mM urea solutions with different NaCl concentrations (0.05, 0.5, 5, 25 and 50 mM), analyzed by tracking 20 particles. (b) The diffusion coefficient of the nanomotors at each condition was determined. Ionic self-diffusiophoresis is a plausible explanation, as the ionic species have different diffusivities. These differences between the cations and anions lead to the

generation of local electric fields and double-layer polarization around the particle, initiating its motion. Consequently, increasing the ionic strength in the medium inhibits the formation of a concentration gradient, thereby reducing the self-propulsion of the nanomotors.

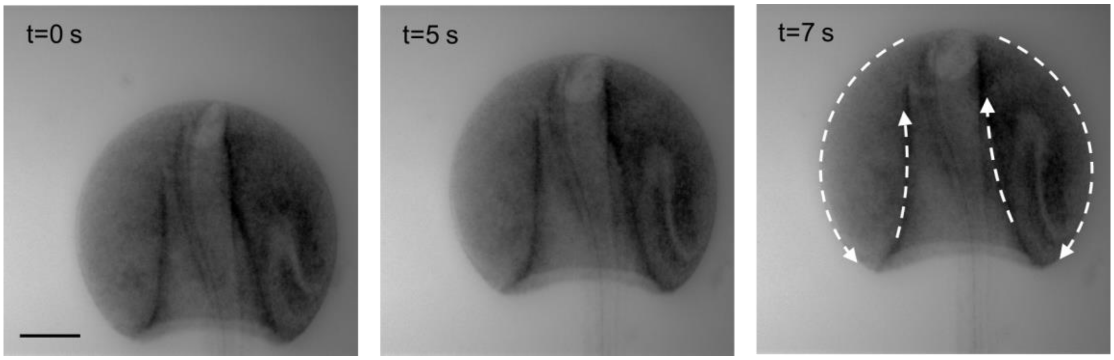

Supplementary Fig. 3 Images of an ascending droplet of UrNMs showing the formation of two counter-rotating vortices, as indicated schematically by the white arrows. The scale bar corresponds to 0.5 mm.

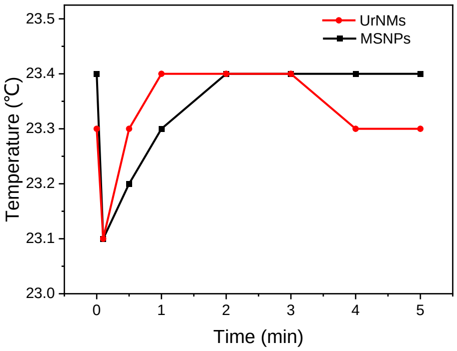

Supplementary Fig. 4 Temperature variation upon the addition of UrNMs and MSNPs.

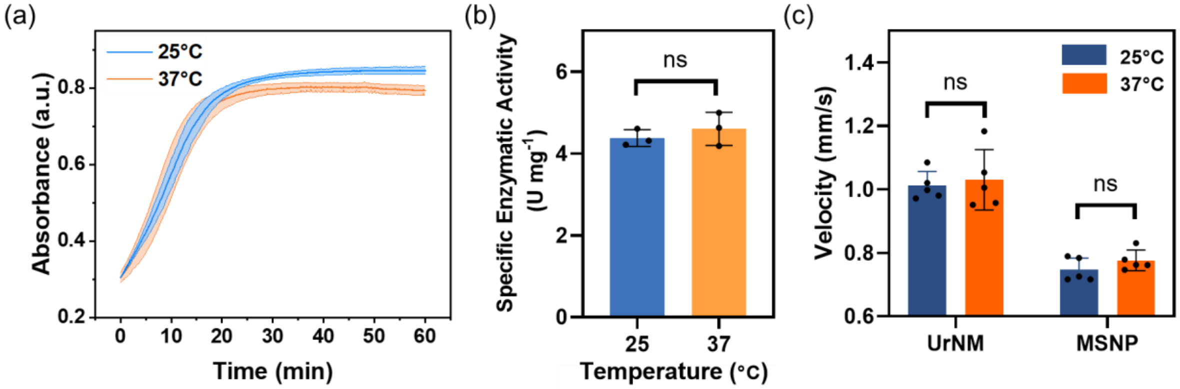

Supplementary Fig. 5 The impact of physiological temperature on enzymatic activity and the collective movement of UrNMs. (a) The real-time UV-vis light absorbance of phenol red solutions containing 200 mM urea and UrNMs at 25°C and 37°C. The data are shown as mean ± SD of three independent experiments (N=3). (b) The specific enzymatic activity of UrNMs at 25°C and 37°C calculated according to (a). N=3. (c) Velocity analysis of active UrNMs swarms and passive MSNPs particulates in 200 mM

urea at 25°C and 37 °C, respectively. N=5. Significant difference is analyzed by students' t-test: ns = not significant (P>0.05).

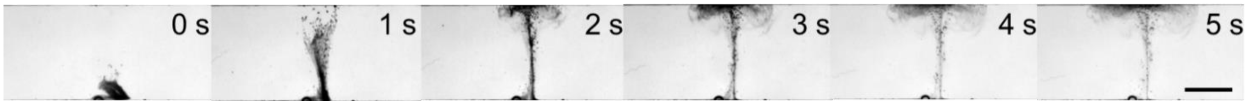

Supplementary Fig. 6 Convective dynamics of CatNMs. A time-lapse sequence of images that show the directional and collective movement of CatNMs in 100 mM H_2O_2. Scale bar: 4 mm.

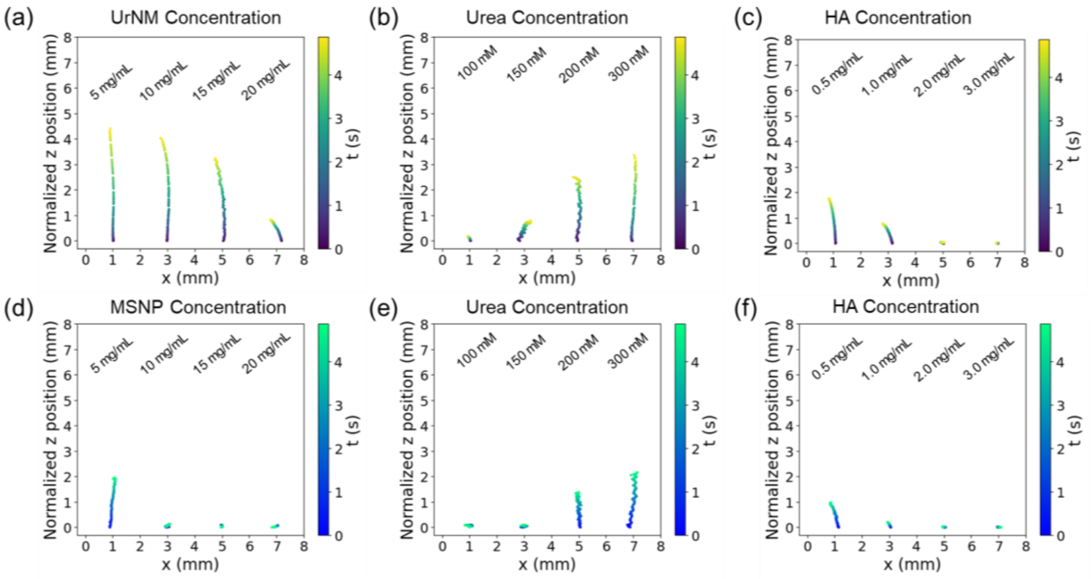

Supplementary Fig. 7 The center of mass tracking of particulates in y-axis within 5 s. The center of mass tracking of UrNMs swarms in y-axis under various conditions: (a) UrNM concentration, (b) urea concentration, and (c) HA concentration. The center of mass tracking of passive MSNP swarms in y-axis under various conditions: (d) MSNP concentration, (e) urea concentration, and (f) HA concentration.

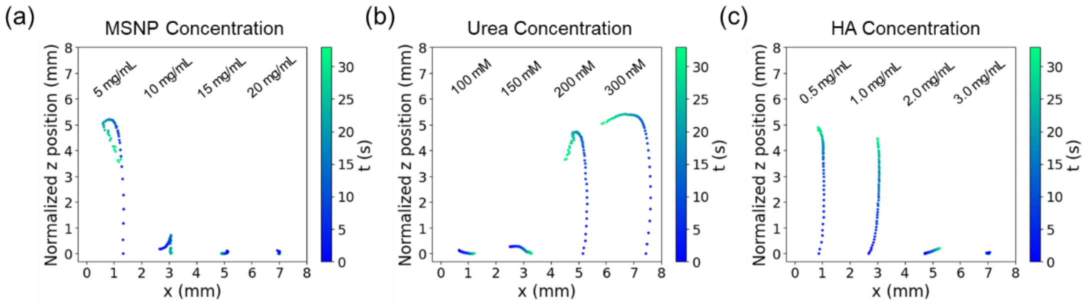

Supplementary Fig. 8 The center of mass tracking of particulates in y-axis within 32 s. The center of mass tracking of passive MSNP swarms under various conditions: (a) MSNP concentration, (b) urea concentration, and (c) HA concentration.

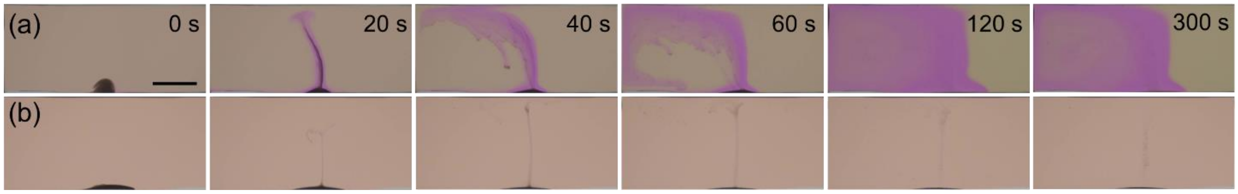

Supplementary Fig. 9 Collective motion of UrNMs/MSNPs indicated by colour change. A time-lapse sequence of images shows the directional and collective movement of (a) UrNMs in urea with phenol red and (b) MSNPs in urea with phenol red. Scale bar: 4 mm.

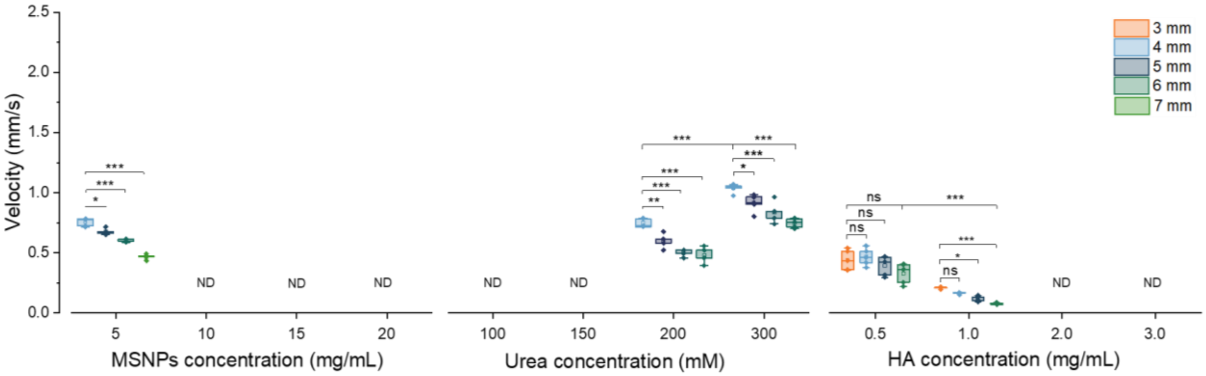

Supplementary Fig. 10 Velocity analysis of passive MSNPs in varied particle concentration, urea concentration, and fuel with diverse HA concentration. The box represents the interquartile range, the whiskers represent the minimum and maximum values, and the central line represents the median. Significant differences were calculated using students' t-test: ***=P < 0.001; **=P < 0.01; *=P < 0.05; ns = not significant (P > 0.05). N=5. ND means velocity is lower than detectable value.

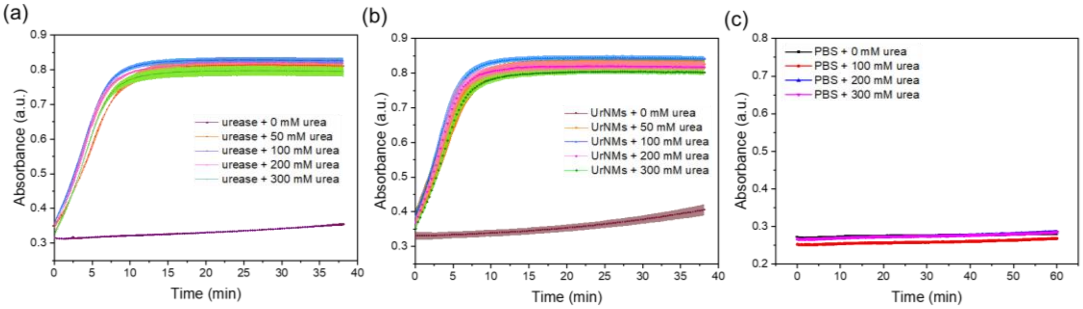

Supplementary Fig. 11 Enzymatic activity measurement in urea dissolved in PBS buffer. The real-time UV-vis light absorbance of phenol red solutions containing varied concentrations of urea after adding (a) urease, (b) UrNMs, or (c) PBS buffer.

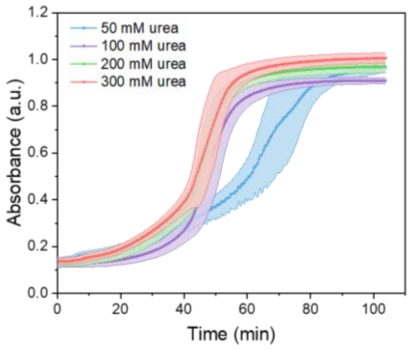

Supplementary Fig. 12 The real-time UV-vis light absorbance of phenol red solutions containing varied concentrations of urea (in acetate buffer) after adding UrNMs. N=3.

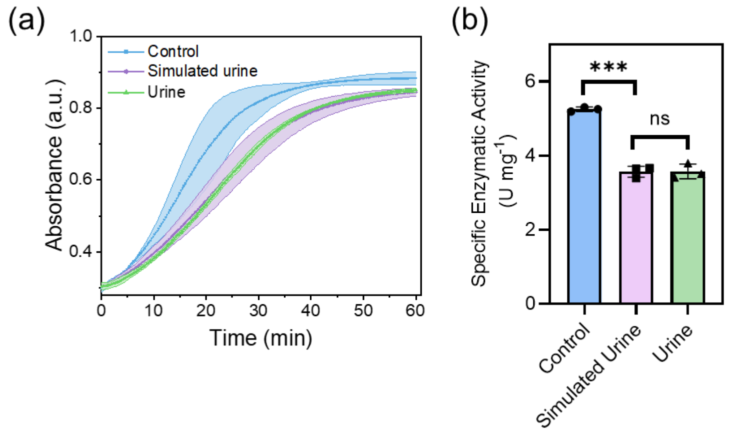

Supplementary Fig. 13 Enzymatic activity of UrNMs after treatment in simulated urine or urine from mice for 30 min. (a) The real-time UV-vis light absorbance of phenol red solutions containing 200 mM urea and treated UrNMs. In the control group, the UrNMs were mixed with PBS solution. (b) The specific enzymatic activity of UrNMs after treatment. Significant difference is analyzed by students' t-test: ***=P < 0.001; ns = not significant (P>0.05). N=3.

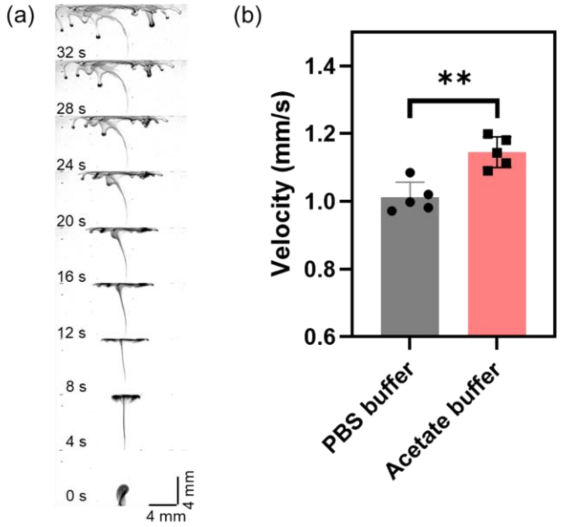

Supplementary Fig. 14 Collective movement of UrNMs in 200 mM urea that is dispersed in acetate buffer. (a) A time-lapse sequence of images that show the collective movement of UrNMs in 200 mM urea in acetate buffer. (b) Velocity analysis of UrNMs particulate in PBS buffer and in acetate buffer. The significant difference is analyzed by student's t-test: **=P < 0.01. N=5.

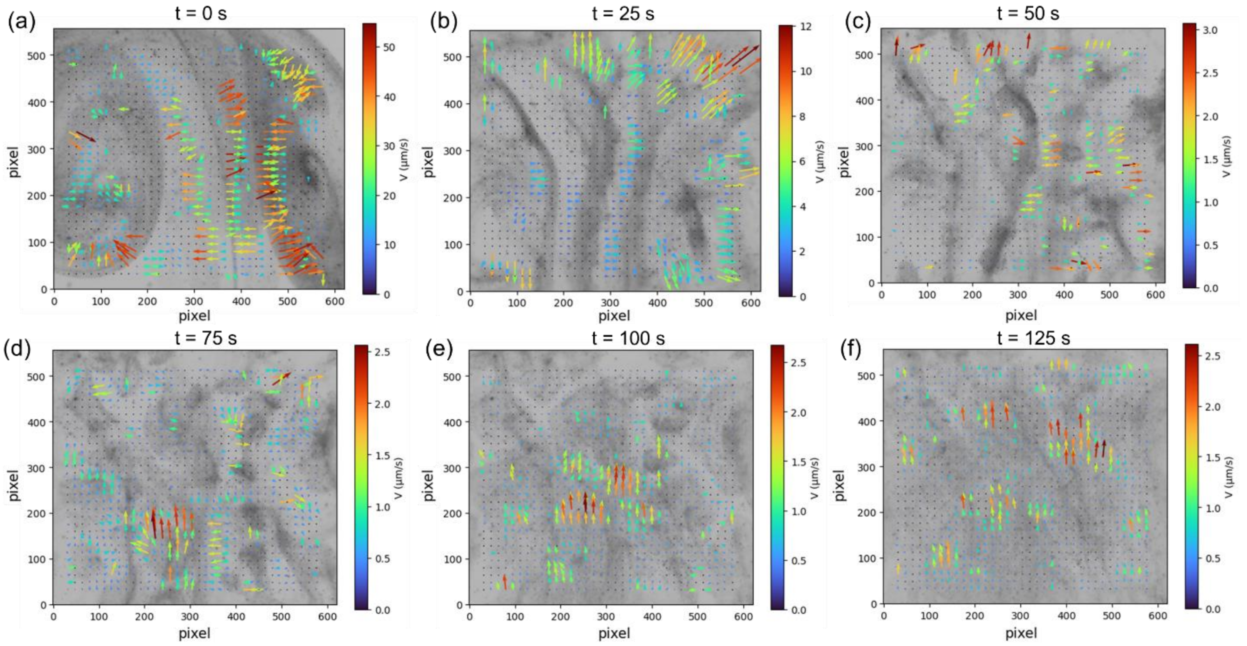

Supplementary Fig. 15 PIV analysis of UrNMs' collective motion in a 1.6 mm-high confinement filled with urea. (a)-(f) are results at different time points.

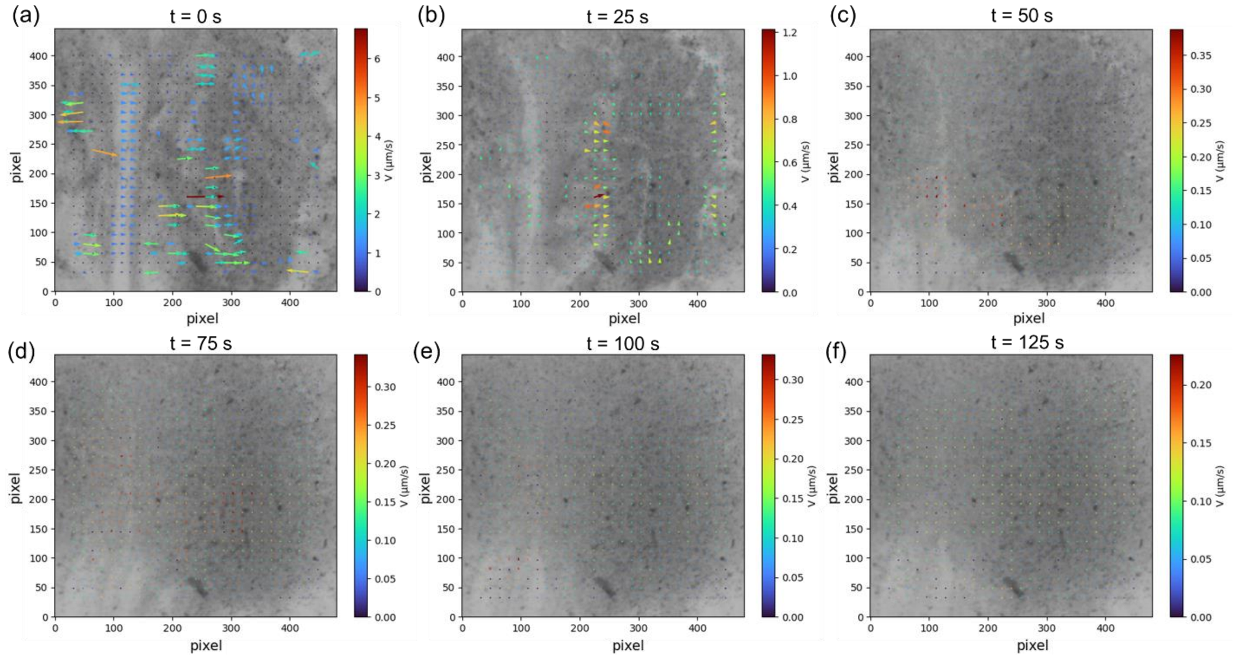

Supplementary Fig. 16 PIV analysis of UrNMs' collective motion in a 0.5 mm-high confinement filled with urea. (a)-(f) are results at different time points.

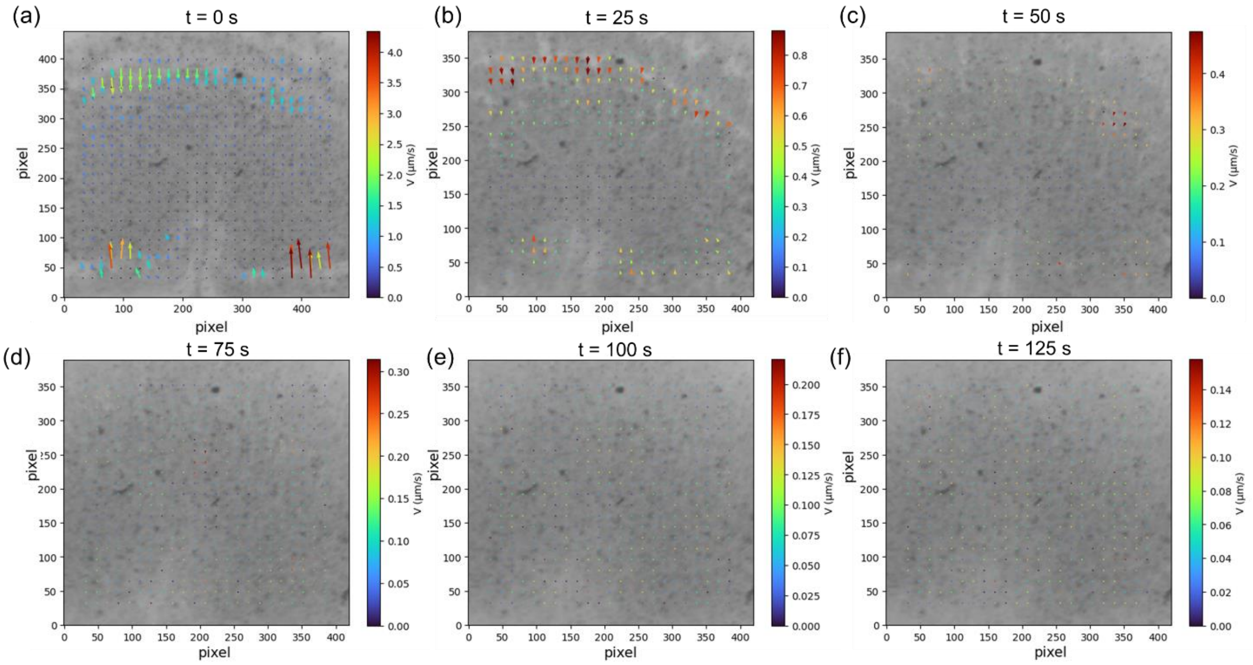

Supplementary Fig. 17 PIV analysis of UrNMs' collective motion in a 0.25 mm-high confinement filled with urea. (a)-(f) are results at different time points.

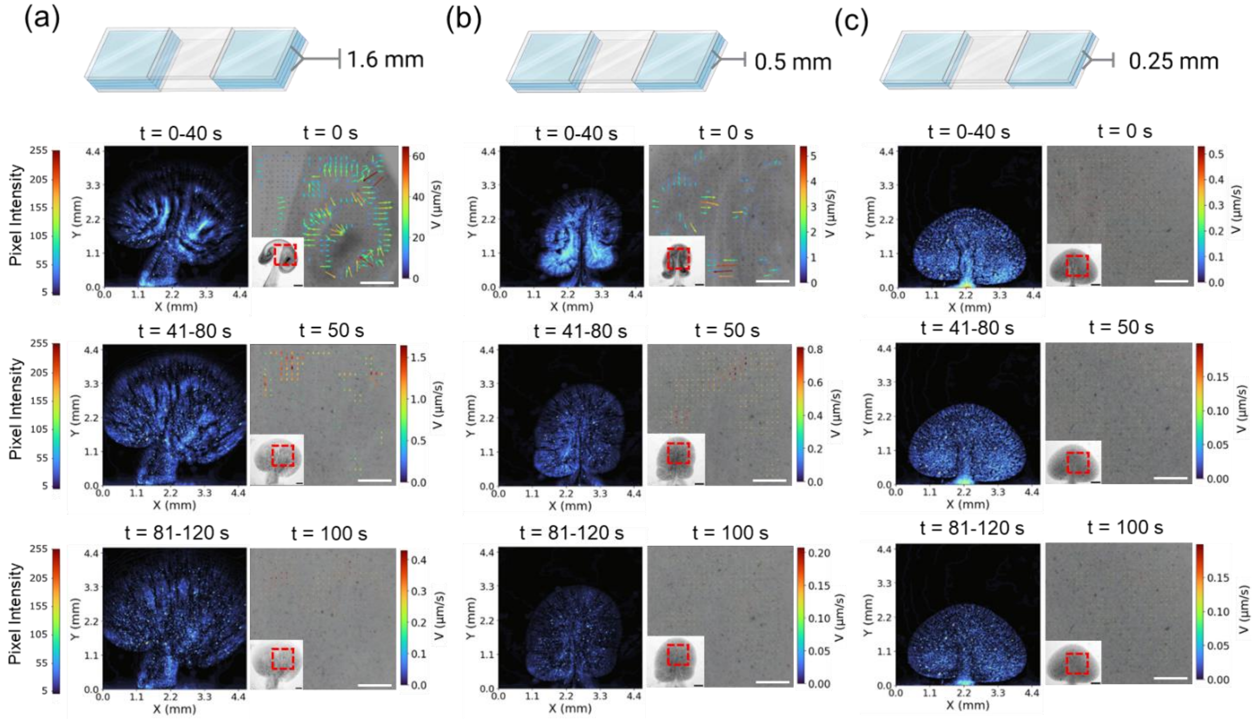

Supplementary Fig. 18 Collective behaviour shaped by vertical confinement. Intensity maps and particle image velocimetry (PIV) analysis are conducted on the collective behaviour of UrNMs in (a) a 1.6 mm-high confinement filled with PBS, (b) a 0.5 mm-high confinement filled with PBS, and (c) a 0.25 mm-high confinement filled with PBS. The average pixel intensity is calculated over 40-second periods from 120-second video recordings (left panels in each figure). The right panels present zoomed-out images that

are snapshots of those video recordings at fixed time points, while the zoomed-in views display corresponding PIV measurements. Scale bars in small panel: 1 mm, in enlarged panel: 0.5 mm. The schematics were created in BioRender. Sánchez, S. (2023) BioRender.com/i44j104.

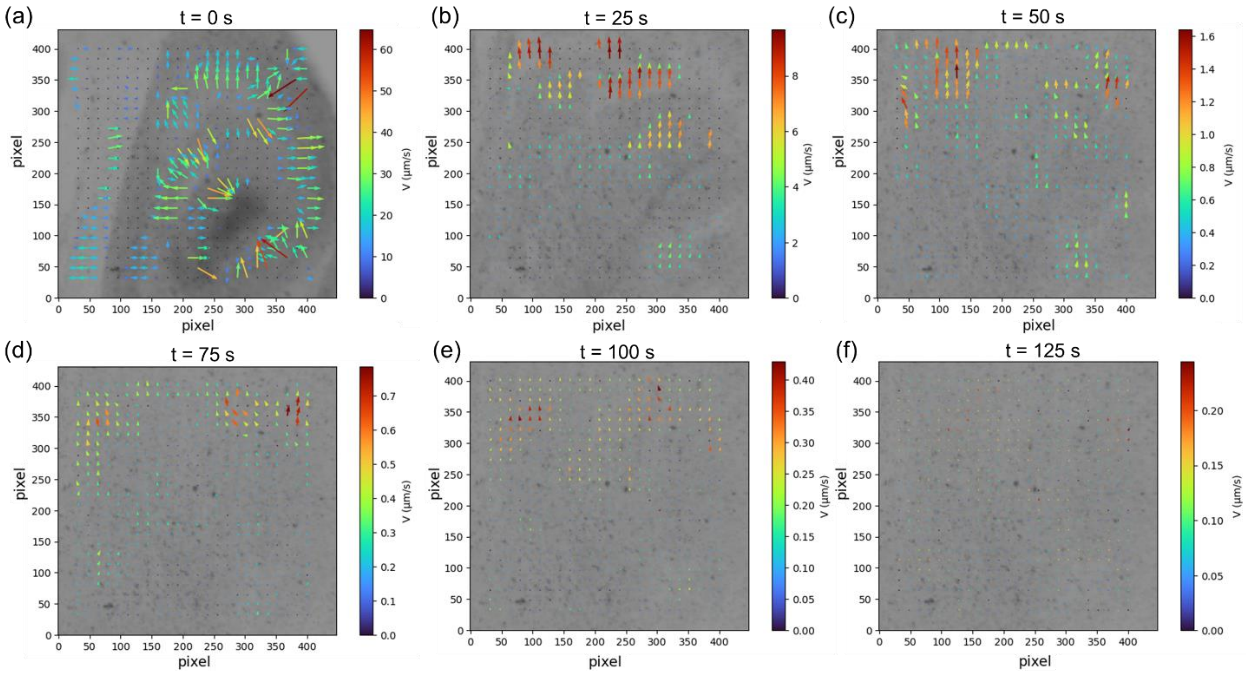

Supplementary Fig. 19 PIV analysis of UrNMs' collective motion in a 1.6 mm-high confinement filled with PBS. (a)-(f) are results at different time points.

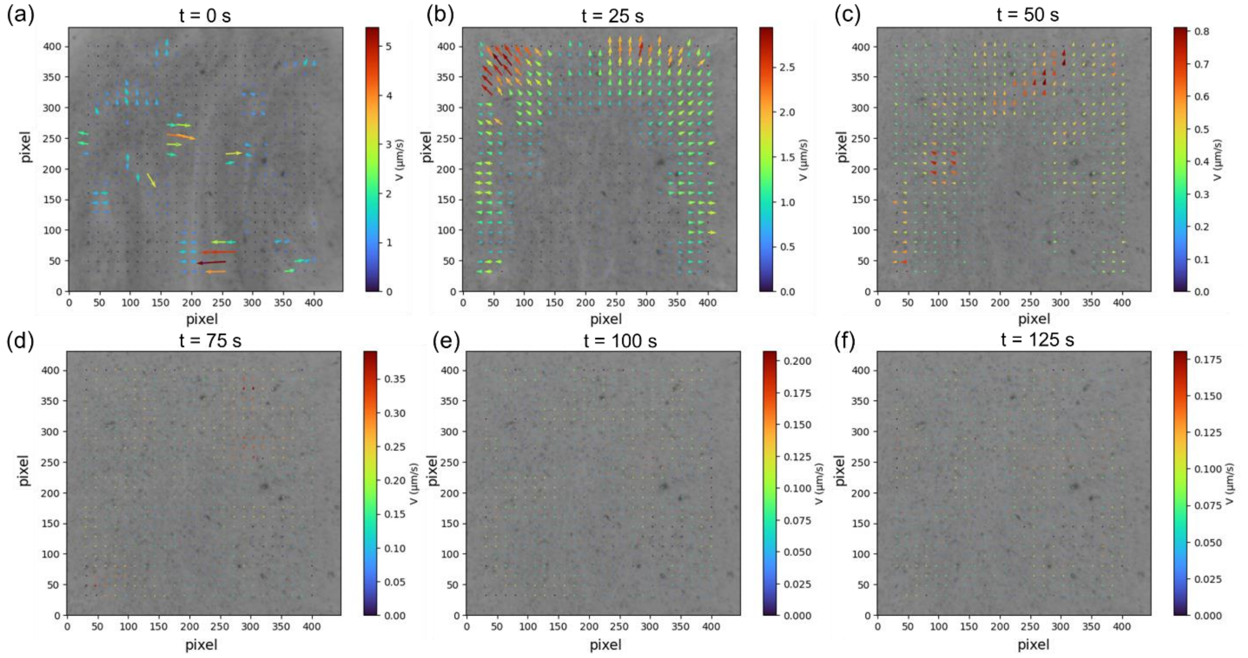

Supplementary Fig. 20 PIV analysis of UrNMs' collective motion in a 0.5 mm-high confinement filled with PBS. (a)-(f) are results at different time points.

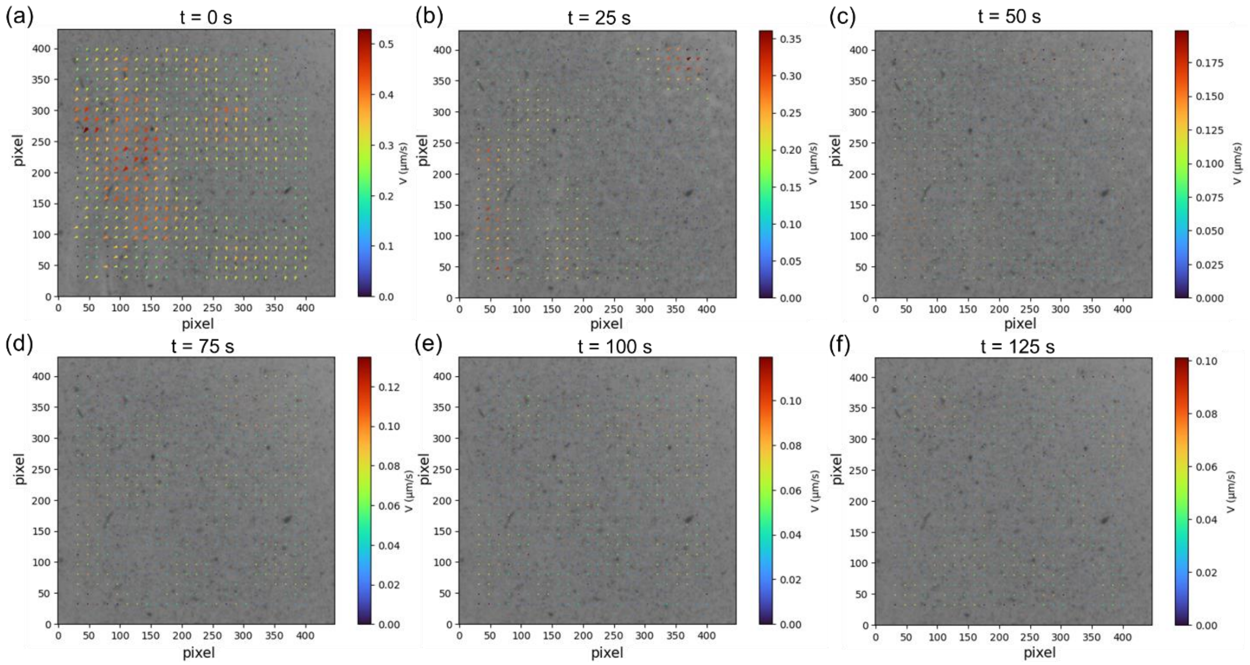

Supplementary Fig. 21 PIV analysis of UrNMs' collective motion in a 0.25 mm-high confinement filled with PBS. (a)-(f) are results at different time points.

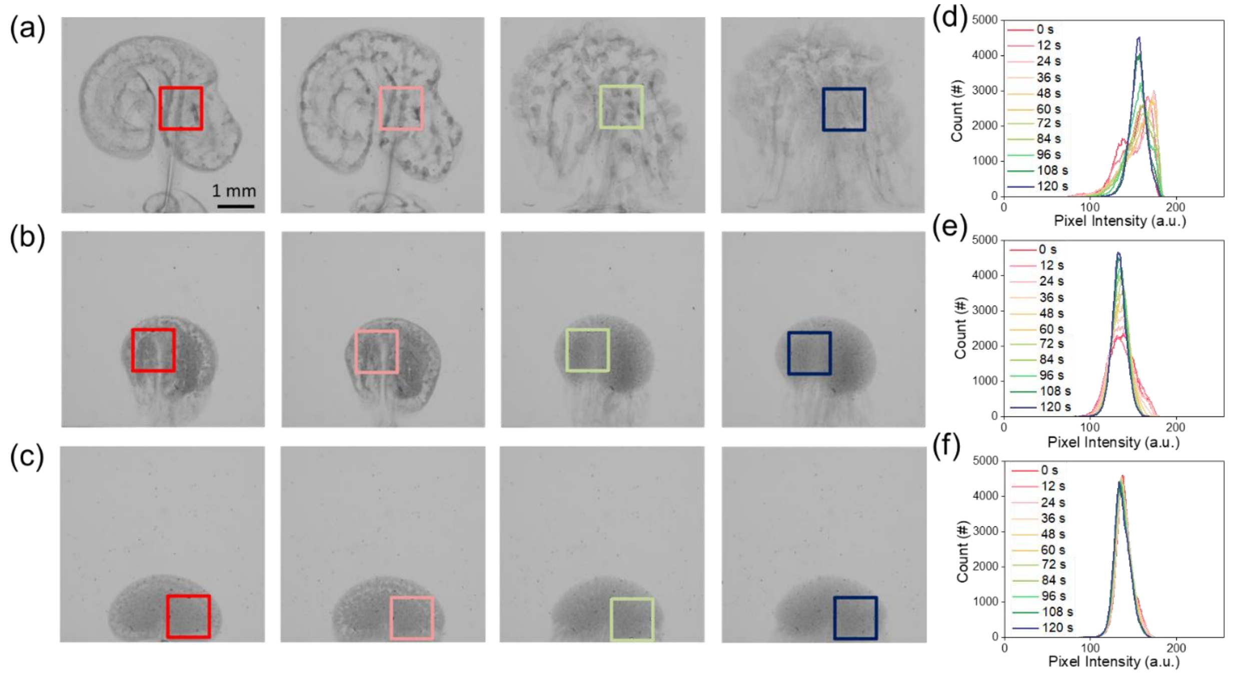

Supplementary Fig. 22 Histograms of pixel intensity distribution within the selected region of interest. Snapshots of UrNMs swarms in vertically confined chamber (filled with fuel) with different heights: (a) 1.6 mm, (b) 0.5 mm, and (c) 0.25 mm, and corresponding pixel intensity distribution histograms (d), (e) and (f) within the region of interest, respectively.

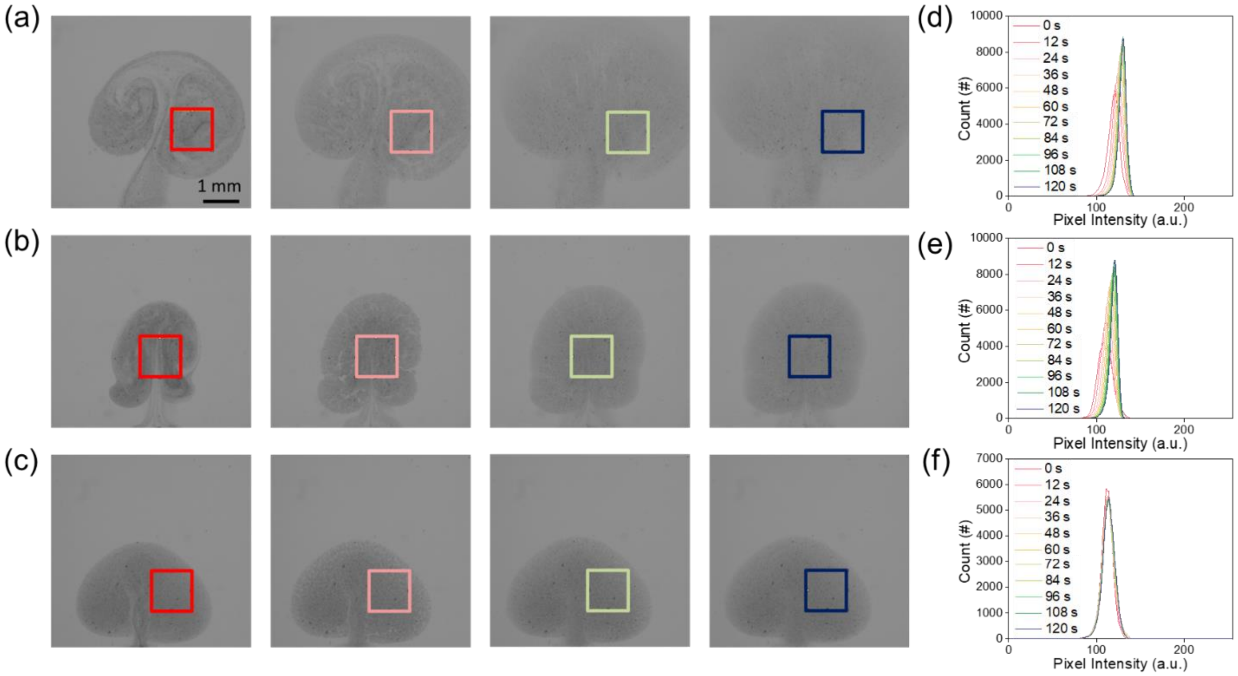

Supplementary Fig. 23 Histograms of pixel intensity distribution within the selected region of interest. Snapshots of UrNMs swarms in PBS solutions that were confined within spaces of different heights: (a) 1.6 mm, (b) 0.5 mm, and (c) 0.25 mm, and corresponding pixel intensity distribution histograms (d), (e) and (f) within the region of interest, respectively.

Supplementary Table 1 The main reactions and rate constants for CO_2 and NH_3 in PBS buffer and acetate buffer.

	Main chemical reactions	Rate constant	Reverse rate constant
In PBS buffer	1. CO_2 (aq) + $OH^- \rightleftharpoons HCO_3^-$	[3]1.21×10^4 $M^{-1}s^{-1}$	[3]4.0×10^{-4} s^{-1}
	2. $HCO_3^- + OH^- \rightleftharpoons CO_3^{2-} + H_2O$	[4]6.0×10^9 $M^{-1}s^{-1}$	[4]3.0×10^5 s^{-1}
	3. CO_2 (aq) + $NH_3 \rightleftharpoons NH_2COOH$	[5]4.5×10^2 $M^{-1}s^{-1}$	[5]68 s^{-1}
In acetate buffer	4. $CO_2 + H_2O \rightleftharpoons HCO_3^- + H^+$	[3]0.037 s^{-1}	[3]1.24×10^5 $M^{-1}s^{-1}$
	5. $HCO_3^- \rightleftharpoons H^+ + CO_3^{2-}$	[4]59.4 s^{-1}	[4]5.0×10^{10} $M^{-1}s^{-1}$
	6. $NH_3 + H^+ \rightleftharpoons NH_4^+$	[6]4.3×10^{10} $M^{-1}s^{-1}$	[6]24.6 s^{-1}
	7. $H^+ + OH^- \rightleftharpoons H_2O$	[4]2.31×10^{10} $M^{-1}s^{-1}$	[4]1.4×10^{-3} M s^{-1}

Supplementary Table 2 The amount of urease attached to the surface of MSNPs by BCA.

Sample number	Initial enzyme added (μg/mL)	Supernatant 1 (μg/mL)	Supernatant 2 (μg/mL)	enzyme attatched (μg/mL)
Urease 1	255.8	151.8	0	104
Urease 2	263.8	151.8	0	112
Urease 3	282.8	156.8	0	126
Catalase 1	380.0	172.7	0	207.3
Catalase 2	376.4	187.3	0	189.1
Catalase 3	430.9	205.4	0	225.5

References

[1] Aranson, I. S. & Sapozhnikov, M. V. Theory of pattern formation of metallic microparticles in poorly conducting liquids. *Phys. Rev. Lett.* **92**, 234301-234304 (2004).

[2] Sapozhnikov, M. V., Tolmachev, Y. V., Aranson, I. S., & Kwok, W.-K. Dynamic self-assembly and patterns in electrostatically driven granular media. *Phys. Rev. Lett.* **90**, 114301-114304 (2003).

[3] Wang, X. et al. Comprehensive study of the hydration and dehydration reactions of carbon dioxide in aqueous solution. *Phys. Chem. A* **114**, 1734-1740 (2010).

[4] Feng, Y. et al. Self-adaptive enzyme-powered micromotors with switchable propulsion mechanism and motion directionality. *Appl. Phys. Rev.* **8**, 011406-011415 (2021).

[5] Wang, X. et al. Kinetics of the reversible reaction of CO_2 (aq) with ammonia in aqueous solution. *J. Phys. Chem. A* **115**, 6405–6412 (2011).

[6] Emerson, M. T., Grunwald, E., Kromhout, R. A. Proton-transfer studies by nuclear magnetic resonance. I. Diffusion control in the reaction of ammonium ion in aqueous acid. *J. Chem. Phys.* **33**, 547-556 (1960).
