# Supplementary material for: Collective buoyancy-driven dynamics in swarming enzymatic nanomotors
Source: Nat Commun. 2024 Oct 29;15:9315. doi: 10.1038/s41467-024-53664-w (PMC11522643; doi:10.1038/s41467-024-53664-w)
Supplement: Supplementary file 2 — Description of Additional Supplementary Files [file 41467_2024_53664_MOESM2_ESM.pdf]

Description of Additional Supplementary Files

Supplementary Movie 1: The universality of the mechanism

Supplementary Movie 2: Control factors influence the collective behaviour of UrNMs

Supplementary Movie 3: Control factors influence the motion of MSNPs

Supplementary Movie 4: The pH change of the collective dynamics

Supplementary Movie 5: Bubbles produced by enzymatic catalysis reaction

Supplementary Movie 6: Vertical confinement shapes collective behaviour

Supplementary Movie 7: Computational modelling of the control factors

Supplementary Movie 8: Computational modelling of the vertical confinement effects
